# Validation of ICD-9-CM coding algorithm for improved identification of hypoglycemia visits

**DOI:** 10.1186/1472-6823-8-4

**Published:** 2008-04-01

**Authors:** Adit A Ginde, Phillip G Blanc, Rebecca M Lieberman, Carlos A Camargo

**Affiliations:** 1 Division of Emergency Medicine, University of Colorado Denver School of Medicine, Aurora, CO, USA; 2 Division of Emergency Medicine, Beth Israel Deaconess Medical Center, Boston, MA, USA; 3 Division of Emergency Medicine, Massachusetts General Hospital, Boston, MA, USA

## Abstract

**Background:**

Accurate identification of hypoglycemia cases by *International Classification of Diseases, Ninth Revision, Clinical Modification *(ICD-9-CM) codes will help to describe epidemiology, monitor trends, and propose interventions for this important complication in patients with diabetes. Prior hypoglycemia studies utilized incomplete search strategies and may be methodologically flawed. We sought to validate a new ICD-9-CM coding algorithm for accurate identification of hypoglycemia visits.

**Methods:**

This was a multicenter, retrospective cohort study using a structured medical record review at three academic emergency departments from July 1, 2005 to June 30, 2006. We prospectively derived a coding algorithm to identify hypoglycemia visits using ICD-9-CM codes (250.3, 250.8, 251.0, 251.1, 251.2, 270.3, 775.0, 775.6, and 962.3). We confirmed hypoglycemia cases by chart review identified by candidate ICD-9-CM codes during the study period. The case definition for hypoglycemia was documented blood glucose 3.9 mmol/l or emergency physician charted diagnosis of hypoglycemia. We evaluated individual components and calculated the positive predictive value.

**Results:**

We reviewed 636 charts identified by the candidate ICD-9-CM codes and confirmed 436 (64%) cases of hypoglycemia by chart review. Diabetes with other specified manifestations (250.8), often excluded in prior hypoglycemia analyses, identified 83% of hypoglycemia visits, and unspecified hypoglycemia (251.2) identified 13% of hypoglycemia visits. The absence of any predetermined co-diagnosis codes improved the positive predictive value of code 250.8 from 62% to 92%, while excluding only 10 (2%) true hypoglycemia visits. Although prior analyses included only the first-listed ICD-9 code, more than one-quarter of identified hypoglycemia visits were outside this primary diagnosis field. Overall, the proposed algorithm had 89% positive predictive value (95% confidence interval, 86–92) for detecting hypoglycemia visits.

**Conclusion:**

The proposed algorithm improves on prior strategies to identify hypoglycemia visits in administrative data sets and will enhance the ability to study the epidemiology and design interventions for this important complication of diabetes care.

## Background

Hypoglycemia is an important complication in patients with diabetes that has a profound impact on quality of life and self-management. While tight glycemic control remains a hallmark to lower rates of complications [1-3], the barrier of hypoglycemia is the major limiting factor in the maintenance of euglycemia [4]. Mild or self-treated episodes of hypoglycemia are common, especially in type 1 diabetes, with reported rates of two episodes per week [5]. Severe hypoglycemia, or episodes requiring external assistance, may occur at least once a year, and are a significant cause of morbidity [5-7].

Although some episodes of severe hypoglycemia are treated at home by administration of oral glucose or intramuscular glucagon by family members, the most severe episodes require ambulance or emergency department (ED) visits. Although ED visits for severe hypoglycemia represent a small percentage of the total episodes of hypoglycemia in diabetes, they do serve as a good epidemiological marker of the complication and result in significant economical and psychological costs [8].

Most data on the epidemiology of hypoglycemia are based on highly selected patients in the setting of large clinical trials [1-3,7], and may not generalize to the general population. Accurate identification of hypoglycemia cases by *International Classification of Diseases, Ninth Revision, Clinical Modification *(ICD-9-CM) codes may help to describe the epidemiology, monitor trends, and propose interventions to improve diabetes care through national administrative databases and local chart reviews [9]. There are several ICD-9-CM coding options for hypoglycemia, some of which may represent other diagnoses. For example, diabetes with other specified manifestations (250.8) may represent hypoglycemia or diabetic lower extremity ulcers. While several prior studies have utilized ICD-9-CM codes to identify cases of hypoglycemia [10-14], only Johnson et al. described the accuracy of ICD-9-CM codes to identify hypoglycemia episodes, but with significant methodological limitations [10].

Building on this prior work, we evaluated the accuracy a new ICD-9-CM coding algorithm to identify cases of hypoglycemia presenting to the ED. We hypothesized that our proposed algorithm would identify hypoglycemia with a positive predictive value (PPV) of at least 80%.

## Methods

### Study design

This was a multicenter, retrospective cohort study using a structured medical record review for validation of a prospectively derived ICD-9 coding algorithm. We obtained ethics approval with a waiver of informed consent from the Partners Human Research Committee and the Beth Israel Deaconess Medical Center Committee on Clinical Investigation.

### Study setting and population

This study was conducted at three urban, academic EDs, which are active participants in the Emergency Medicine Network [15]. The EDs have a combined annual visit volume of 175,000 and are staffed by emergency medicine, internal medicine, and surgery residents, and patient care is supervised by attending emergency physicians 24 hours/day.

### Study Protocol

Using the electronic medical records system at each site, we searched the following ICD-9-CM codes to identify possible visits for hypoglycemia: 250.3 (diabetes with other coma), 250.8 (diabetes with other specified manifestations) 251.0 (hypoglycemic coma), 251.1 (other specified hypoglycemia), 251.2 (hypoglycemia, unspecified), 270.3 (leucine-induced hypoglycemia), 775.0 (hypoglycemia in an infant born to a diabetic mother), 775.6 (neonatal hypoglycemia), and 962.3 (poisoning by insulins and antidiabetic agents).

Given the diversity of potential ICD-9-CM codes, we searched this broad range of codes and in all diagnosis fields (up to ten listed) in an attempt to capture all possible ED hypoglycemia visits. For admitted patients, we examined only ED-based codes, to avoid inclusion of incident hypoglycemia that occurred during inpatient hospitalization. In cases where multiple candidate codes were present, we recorded only the first-listed code. The exception to this was for the more ambiguous codes 250.3 and 250.8, for which we preferentially recorded any of the other candidate codes if present. We based this strategy on detailed examination of the ICD-9-CM coding manual [9], review of the experience from previously reported approaches [10-14], and discussion with coding experts.

**Figure 1:**
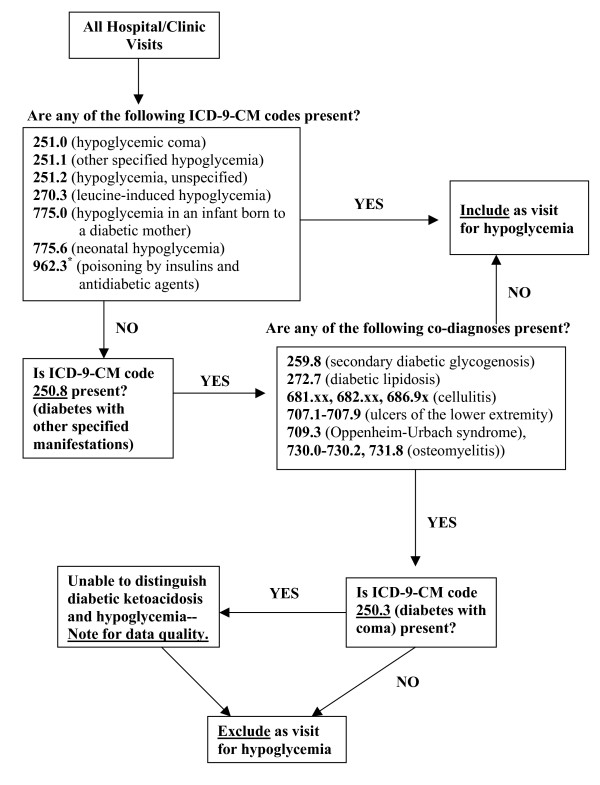
**ICD-9-CM coding algorithm to identify emergency department visits for hypoglycemia**. ICD-9-CM – International Classification of Diseases, Ninth Revision. * Consider exclusion of this code from algorithm, since positive predictive value was 54% in this analysis, and exclusion improved the accuracy of the algorithm.

The code 250.8 may be used for other specific diabetes-associated complications in addition to hypoglycemia, including: 259.8 (secondary diabetic glycogenosis), 272.7 (diabetic lipidosis), 707.xx (ulcers of the lower extremity), 709.3 (Oppenheim-Urbach syndrome), and 730.0–730.2, 731.8 (osteomyelitis). Based on discussion with coding experts, we determined that 681.xx (cellulitis of fingers/toes), 682.xx (other cellulitis), and 686.9x (local skin infection) may also be utilized as a co-diagnoses for 250.8, although not specifically mentioned in the manual. We prospectively proposed the coding algorithm displayed in Figure 1 and validated its accuracy through chart review.

We identified all ED visits with candidate ICD-9-CM codes between July 1, 2005 and June 30, 2006 at each site, and obtained written ED charts. For patients with multiple ED visits during the data collection period, we requested only the first visit to avoid overrepresentation by certain patients. Trained research staff abstracted all charts using a standardized form, and the research group met weekly to discuss data collection and resolve abstraction issues. Additionally, two reviewers independently abstracted 10% of charts to evaluate inter-rater agreement in data collection. To enhance the reliability of our chart review, we abstracted only charts with complete ED triage assessment, nursing notes, and emergency physician notes and considered all other charts incomplete.

### Key Outcome Measures

We considered this chart validation as the gold standard for confirmation of true hypoglycemia visits. In reviewing the ED chart, we confirmed cases of hypoglycemia based on the following criteria: 1) Any documented pre-hospital or ED glucose value (serum or capillary) 3.9 mmol/l, or 2) Charted physician discharge diagnosis of hypoglycemia. We based the glucose threshold on the consensus recommendation defined by the American Diabetes Association Workgroup on Hypoglycemia [16]. We included physician diagnosis of hypoglycemia to capture cases in which hypoglycemia resolved prior to first glucose level, i.e. patients receiving glucose for symptoms consistent with hypoglycemia prior to blood glucose determination. Additionally, we collected patient disposition (discharge or hospital admission) to evaluate for differences in coding accuracy based on this factor.

### Data analysis

We performed statistical analyses using Stata 9.0 (College Station, TX). We summarized data using basic descriptive statistics. We determined accuracy of specific codes and coding algorithm by calculating PPV with 95% confidence intervals (CI). Although we included a broad range of possible ICD-9-CM codes, the numbers of missed hypoglycemia visits, not captured by the candidate codes, were unknown. Under the ideal assumption that no cases were missed, we calculated estimated point estimates for sensitivity, specificity, and negative predictive values (NPV). Additionally, we performed a sensitivity analysis by increasing the presumed number of missed cases by 10%.

We determined inter-rater agreement for chart abstraction by calculating the kappa statistic for the subgroup of charts abstracted by two investigators. Additionally, we compared accuracy of the algorithm stratified by candidate ICD-9-CM code position, study site and ED disposition using chi-squared test and with two-tailed p < 0.05 considered statistically significant. Finally, we evaluated the accuracy of all ICD-9-CM codes to identify potential improvements and calculated the accuracy of the revised algorithm including the proposed modifications.

## Results

Of the 174,134 ED visits at the three institutions during the data collection period, we identified 901 patients with candidate ICD-9-CM codes. These patients accounted for 1,139 visits with possible hypoglycemia (i.e., 0.5% of all ED visits). We abstracted 679 charts (75% of visits by unique patients) and excluded the remaining 222 patients with missing or incomplete documentation. There were no significant differences in age, sex, race/ethnicity, and distribution of candidate ICD-9-CM codes comparing abstracted and excluded patients (all p > 0.05).

Based on the chart review, we confirmed 436 (64%) cases of hypoglycemia. Confirmation of hypoglycemia had a very high inter-rater agreement (kappa = 0.97). Predictive values for individual ICD-9-CM codes are presented in Table 1. The majority (83%) of confirmed cases of hypoglycemia were coded as 250.8.

**Table 1:** Predictive value of ICD-9-CM codes for identification of hypoglycemia

ICD-9-CM Code	n (%)	Confirmed Cases (%)	PPV (95%CI)
250.30	1 (0.2)	1 (0.2)	100%
250.8x	586 (86)	363 (83)	62% (58–66)
Co-diagnoses absent*	385 (57)	353 (81)	92% (88–94)
251.10	3 (0.4)	2 (0.5)	67% (9–99)
251.20	64 (9)	56 (13)	88% (77–94)
775.60	1 (0.2)	1 (0.2)	100%
962.30	24 (4)	13 (3)	54% (32–74)
Algorithm^+^	477 (70)	425 (97)	89% (86–92)
Total	679	436	64% (60–68)

ICD-9-CM – International Classification of Diseases, Ninth Revision; n – number; CI – confidence interval; PPV – positive predictive value.

* Co-diagnoses are: 259.8, 272.7, 681.xx, 682.xx, 686.9x, 707.xx, 709.3, 730.0–730.2, and 731.8.

+ Predictive value of proposed algorithm (see Figure 1)

Table 2 shows the accuracy of excluding hypoglycemia cases for visits with ICD-9-CM code 250.8 using the proposed alternate co-diagnoses. The absence of any co-diagnosis codes improved the positive predictive value of 250.8 from 62% to 92%, while excluding 10 (2%) of true hypoglycemia visits. These false-negative cases occurred when either cellulitis (681.xx, 682.xx, 686.9x) or ulcers (707.xx) were present concurrently with hypoglycemia.

**Table 2:** Accuracy of co-diagnosis codes to exclude hypoglycemia for ICD-9-CM code 250.8

ICD-9-CM code*	n (%)	Valid exclusion (%)	PPV (95%CI)
681.xx (cellulitis fingers/toes)	9 (4)	9 (5)	100%
682.xx (other cellulitis)	86 (43)	78 (41)	91% (83–96)
686.90 (local skin infection)	6 (3)	6 (3)	100%
707.xx (ulcers of lower limb)	92 (46)	90 (47)	98% (94–100)
730.27 (osteomyelitis ankle/foot)	2 (1)	2 (1)	100%
731.8 (other osteomyelitis)	6 (3)	6 (3)	100%
Total	201	191	95% (91–98)

ICD-9-CM – International Classification of Diseases, Ninth Revision; n – number; CI – confidence interval; PPV – positive predictive value.

* No observations for 259.8 (secondary diabetic glycogenosis), 272.7 (diabetic lipidosis), or 709.3 (Oppenheim-Urbach syndrome)

Overall, the proposed algorithm had PPV of 89% (95%CI, 86–92) for detecting hypoglycemia visits. There was no significant difference in PPV when data were stratified by site or by ED disposition (p = 0.86 and 0.22, respectively). Based on the assumption that the candidate ICD-9-CM codes captured all ED presentations with hypoglycemia, we estimate that the algorithm had 97% sensitivity, >99% specificity, and >99% NPV. Assuming the algorithm missed at most 10% of hypoglycemia visits, we estimate a minimum sensitivity of 88% with NPV and specificity remaining >99%.

Table 3 presents the relationship between ICD-9-CM code position and confirmed hypoglycemia cases, stratified by the common candidate codes. Over a quarter of confirmed cases had a candidate code outside of the first-listed diagnosis field. Algorithm codes in the first ICD-9-CM position had a higher PPV for confirmed hypoglycemia visits than other positions (93% vs. 81%, p < 0.01).

**Table 3:** Association between diagnostic code position and identification of hypoglycemia

	Total confirmed hypoglycemia cases				
ICD-9-CM position	n (%)	250.8x	251.20	962.30	Other
1^st^	319 (73)	287	18	12	2
2^nd^	85 (19)	55	29	0	1
3^rd^	21 (5)	14	6	0	1
4^th ^or higher	11 (3)	7	3	1	0
Total	436	363	56	13	4

ICD-9-CM – International Classification of Diseases, Ninth Revision

Post-hoc review of the algorithm performance suggested that cases identified by 962.3 demonstrated low PPV (54%), primarily representing patients with excessive insulin or oral hypoglycemic use without development of clinical or laboratory hypoglycemia. We evaluated the full list of ICD-9-CM codes for all visits coded as 962.3 and found that 10 of 13 confirmed cases of hypoglycemia had 250.8 listed as a co-diagnosis and only 1 of 11 cases without hypoglycemia had 250.8 as a co-diagnosis. When we removed 962.3 as a candidate code and relied only on coding for 250.8 in these cases, the PPV for the overall algorithm increased from 89% to 91% (95%CI, 88–93) while the estimated sensitivity remained at 97%.

## Discussion

In this study, we demonstrated high predictive value for detection of ED hypoglycemia visits using the new ICD-9-CM coding algorithm. To our knowledge, this is the first chart validation of the accuracy of ICD-9-CM codes for hypoglycemia, an important complication in diabetes and common ED presentation.

Our algorithm differs in several important ways from prior reports on hypoglycemia. Two separate analyses of the relationship between anti-hypertensive agents and hypoglycemia in patients with diabetes, Herings et al. and Morris et al. used ICD-9-CM codes 251.0, 251.1, and 251.2 to identify possible episodes of hypoglycemia [12,13]. Schorr et al. broadened candidate codes to include 250.3 and 962.3 [11,14]. Notably, none of these studies included 250.8 in their search strategies, nor accounted these possible missed episodes of hypoglycemia. In our analysis, 250.8 comprised the vast majority (83%) of hypoglycemia cases, and exclusion of this code in these prior studies likely contributed to substantial underestimates in the incidence of hypoglycemia.

Johnson, et al. first reported the utility of 250.8 in their strategy to identify ED visits for hypoglycemia [10]. In their analysis, this code also identified over half of hypoglycemia episodes and had a PPV of 73% for their case definition of hypoglycemia. Our chart validation differed in several important ways. First, Johnson et al. limited their search to first-listed ICD-9-CM codes and excluded all other diagnosis fields. We evaluated all diagnosis fields and while candidate codes in the first diagnosis field had a higher PPV, limitation to the primary diagnosis field would have excluded 27% of hypoglycemia cases. Additionally, candidate codes in the latter diagnosis fields maintained a high PPV (81%). Secondly, their validation of hypoglycemia cases involved searching of brief communications following the ED visit for symptoms consistent with hypoglycemia, which included confusion, loss of consciousness, and seizure. While these symptoms are consistent with hypoglycemia, they are nonspecific and have many other causes in addition to hypoglycemia [17]. We performed detailed chart reviews to confirm hypoglycemia visits, which allowed for more precise validation. Finally, we confirmed the importance of 250.8 in identification of the majority of hypoglycemia visits, but additionally refined the search strategy by exclusion of visits with alternate co-diagnoses. Exclusion of these cases reliably eliminated 85% of false-positive cases coded as 250.8, while eliminating only a small number of true-positive cases.

Overall, 250.8 with exclusion of alternate co-diagnoses and 251.2 comprised >95% of hypoglycemia identified by the algorithm. Although there were insufficient data to evaluate 250.0 and 250.1 in this analysis, we recommend inclusion of these codes in any search for hypoglycemia. These codes are likely to have high specificity/PPV, and their utilization may be higher in different institutions and practice environments. As anticipated, codes 270.3, 775.0 and 775.6 were rare in the ED setting but may have utility for other settings, such as maternity and neonatal units.

We hypothesized that we would not be able to determine if diabetes with other coma (250.3) were caused by hypoglycemia or diabetic ketoacidosis (DKA). There were insufficient data to evaluate this hypothesis (only one case), and charts identified with this code should be interpreted with caution. Compared to prior 100% PPV in prior analysis [10], we were surprised by the relatively low predictive value (54%) of 962.3. Based on this finding and our secondary analysis, we recommend exclusion of 962.3 and relying only on 250.8 to identify most of these associated visits. This proposed strategy will require validation in other institutions and settings.

The ICD-9-CM classification system is imperfect for case identification, as it was created for reimbursement rather than research purposes [18]. Given this inherent limitation, the proposed algorithm for hypoglycemia compares favorably to those suggested for a variety of diseases. ICD-9-CM coding accuracy for upper gastrointestinal disorders (93–95% PPV) and Clostridium difficile colitis (87% PPV) were similar [19,20], but were more unreliable for stroke (61–79% PPV) [21], soft tissue disorders (64% PPV) [22], pneumococcal pneumonia (58% PPV) [23], traumatic brain injury (20–38% PPV) [24], and venous thromboembolism (31% PPV) [25]. Based on these comparisons, the proposed ICD-9-CM coding algorithm for hypoglycemia would be expected to perform as well or better than coding strategies for other diagnoses in accurate case identification from administrative data.

### Limitations

The current study has some potential limitations. We did not abstract the 25% of charts that were missing or incomplete. While this lowered the number of total cases, the distribution of ICD-9-CM codes was similar in abstracted and non-abstracted charts, and the likelihood of biased accuracy estimates was small. The coding of hypoglycemia visits was based on three academic EDs and may not generalize to other areas of the hospital, outpatient providers, and other geographic areas. However, there are a finite number of options for coding hypoglycemia, and although the frequency of individual code usage will likely vary based on local practice and familiarity of the coding personnel, we anticipate that accuracy will not vary substantially. We were unable to formally evaluate false-negatives in our data set. Thus, we were only able to calculate PPV with accuracy, and our estimates for sensitivity, specificity, and NPV are significantly limited. To help address this limitation, we performed a sensitivity analysis, which demonstrated a high level of accuracy even when we increased the assumption of missed cases, but inferences based on these values should be guarded. Finally, the accuracy of case confirmation by chart review depended on a retrospective evaluation for hypoglycemia, which may overestimate or underestimate the true number of cases. Standardized definitions and training of reviewer limited the potential for such bias, and high inter-rater agreement demonstrated internal reliability of the chart review.

## Conclusion

We derived and validated a new ICD-9-CM coding algorithm to identify hypoglycemia visits with high predictive validity. This algorithm improves on prior strategies to identify hypoglycemia cases in administrative data sets and will enhance the ability to study the epidemiology and design interventions for this important complication of diabetes care.

## Competing interests

AAG has received investigator-initiated research funding from Novo Nordisk and Bayer in the past 12 months. He does not own stocks or other ownership interest in any company with an interest in diabetes. CAC has received financial support from a variety of groups for participation in conferences, consulting, and medical research. Over the past 12 months, industry sponsors with an interest in diabetes were AstraZeneca, Bayer, GlaxoSmithKline, Merck, Novartis, and Novo Nordisk. He does not own stocks or other ownership interest in any of these companies. The other authors do not have any potential competing interests to report.

## Authors' contributions

All authors participated in study conception and design. AAG participated in data collection, performed the statistical analysis, drafted the manuscript, and takes responsibility for the manuscript as a whole. PGB and RML participated in data collection. CAC provided study supervision and statistical expertise. All authors read the manuscript, participated in critical revision, and approved the final version.

## Pre-publication history

The pre-publication history for this paper can be accessed here:


